# HIV-2 diversity displays two clades within group A with distinct geographical distribution and evolution

**DOI:** 10.1093/ve/veab024

**Published:** 2021-03-16

**Authors:** Benoit Visseaux, Mélanie Bertine, Quentin Le Hingrat, Valentine Ferré, Charlotte Charpentier, Fidéline Collin, Florence Damond, Sophie Matheron, Stéphane Hué, Diane Descamps

**Affiliations:** 1 Université de Paris, IAME, UMR 1137, INSERM, Paris, France; 2 Assistance Publique – Hôpitaux de Paris, Hôpital Bichat, Department of Virology, Paris, France; 3 ISPED, UMR 897, INSERM, Université de Bordeaux, Epidémiologie-Biostatistique, Bordeaux, France; 4 Assistance Publique – Hôpitaux de Paris, Hôpital Bichat, Department of Infectious and Tropical Diseases, Paris, France; 5 Department of Infectious Disease Epidemiology, London School of Hygiene and Tropical Medicine, London, UK

**Keywords:** HIV-2, molecular epidemiology, viral diversity, phylogenetic

## Abstract

Genetic diversity of HIV-2 groups A and B has not yet been fully described, especially in a few Western Africa countries such as Ivory-Coast or Mali. We collected 444 *pol*, 152 *vif*, 129 *env*, and 74 LTR sequences from patients of the French ANRS CO5 HIV-2 cohort completed by 221 *pol*, 18 *vif*, 377 *env*, and 63 LTR unique sequences from public databases. We performed phylogenetic reconstructions and revealed two distinct lineages within HIV-2 group A, herein called A1 and A2, presenting non-negligible genetic distances and distinct geographic distributions as A1 is related to coastal Western African countries and A2 to inland Western countries. Estimated early diversification times for groups A and B in human populations were 1940 [95% higher probability densitiy: 1935–53] and 1961 [1952–70]. A1 experienced an early diversification in 1942 [1937–58] with two distinct early epidemics in Guinea-Bissau or Senegal, raising the possibility of group A emergence in those countries from an initial introduction from Ivory-Coast to Senegal, two former French colonies. Changes in effective population sizes over time revealed that A1 exponentially grew concomitantly to Guinea-Bissau independence war, but both A2 and B lineages experienced a latter growth, starting during the 80s economic crisis. This large HIV-2 genetic analysis provides the existence of two distinct subtypes within group A and new data about HIV-2 early spreading patterns and recent epidemiologic evolution for which data are scarce outside Guinea-Bissau.

## 1. Introduction

Although HIV type 1 (HIV-1) and HIV type 2 (HIV-2) share a common structural and genomic organization, they have different ancestry. HIV-1 descends from simian immunodeficiency viruses (SIV) infecting apes (chimpanzee and gorilla), while HIV-2 ancestors were identified among SIVs infecting Sooty mangabey. The notion of group refers to highly divergent viruses among each of these types that result of distinct cross-species transmissions from apes or monkeys to humans. For some of these groups, the virus has diverged further though founder effects, leading to distinct subtypes and sub-subtypes (Robertson et al. 2000). To date, HIV-2 has been classified into ten distinct groups named A, B, C, D, E, F1, F2, G, H, and I (Gao et al. 1994; Chen et al. 1997; Yamaguchi, Devare, and Brennan. 2000; Damond et al. 2004; Smith et al. 2008; Ayouba et al. 2013). Among them, only groups A and B are endemic and, to date, no subtypes have been formally retained for any HIV-2 group but divergent lineages have been proposed among HIV-2 group A (Breuer et al. 1995; Damond et al. 2001; Visseaux et al. 2016).

The HIV-2 epidemic is concentrated in two main areas of Western Africa. The first one, centred around Ivory Coast and Mali, two former French colonies presenting important HIV-2 epidemics (Visseaux et al. 2016), is caused by both groups A and B and linked to the Taï Forest in Ivory Coast, suggested to be the birthplace of these two groups of HIV-2 (Santiago et al. 2005). The second one, spanning Guinea-Bissau and its neighbouring countries, is composed almost exclusively of HIV-2 group A and represents the largest part of the HIV-2 epidemic (Visseaux et al. 2016). The larger prevalence of HIV-2 in the latter area has been previously hypothesized as linked to the independence war of Guinea-Bissau, occurring from 1963 to 1974 and believed to have led to a massive spread of HIV-2 across among the neighbouring countries but also Portugal and most of its former colonies. This spread led to a HIV-2 prevalence of 9 per cent in Guinea-Bissau in the 90s (Pieniazek et al. 1999; Lemey et al. 2003; Santiago et al. 2005; Visseaux et al. 2016). In more recent years, HIV-2 prevalence in Guinea-Bissau has decreased to 4.7 per cent in 2007 (Fryer et al. 2015).

Today, there are still some uncertainties about HIV-2 diversity, early emergence and epidemic history across Western Africa. Thus, an early work has suggested the existence of three distinct lineages within group A based on a *env* gene analysis of 20 HIV-2 strains (Breuer et al. 1995). In a more recent study, based on a *pol* gene analysis of 30 HIV-2 patients, only two distinct lineages were identified and a distinct geographic distribution within West-Africa was suggested (Damond et al. 2001). The spread of the virus from Ivory Coast to Guinea-Bissau, its suggested epicentre, is also still unclear. Indeed, these two countries had different colonial ties, with only little connections: Portugal for Guinea-Bissau and France for Ivory Coast. Likewise, due to the difficulty to distinguish between HIV-1 and HIV-2 infection using serological testing, recent epidemiological data on HIV-2 prevalence in Mali or Ivory Coast are lacking. Thus, to date, it is not possible to know if the recent HIV-2 decrease observed in Guinea-Bissau is limited to this country or common to all West Africa.

France is the European country with the second highest HIV-2 prevalence, after Portugal. Like Portugal, the higher HIV-2 prevalence in France is explained by its colonial past and the links still present with high prevalence countries in West Africa. The distinctiveness of the French HIV-2 epidemic comes from the inclusion of patients with a wide variety of geographical origins within West Africa, from Senegal at the Western to Mali and Ivory Coast at the Eastern part of Western Africa. Most of the patients diagnosed with HIV-2 in France are included in the Agence Nationale de Recherche sur le Sida et les hépatites virales (ANRS) CO5 HIV-2 cohort, a nationwide database that curates clinical, epidemiological and viral genomic data from more than 1,000 patients living with HIV-2 in France. As 85 per cent of all included patients are born in Western Africa, this database represents a unique opportunity to investigate the early dispersal and diversification of HIV-2 across all West Africa.

In this study, we aimed to confirm and define the existence of several distinct lineages within HIV-2 group A by using a large dataset of sequences and several genome regions. We tested potential geographical or clinical differences among those lineages. We also performed an updated phylogenetic and phylodynamic reconstruction of HIV-2 origins, hypothesizing that the unique geographic diversity of HIV-2-infected patients in France, taken together with all non-French sequences available to date, may provide a balanced and complete picture of HIV-2 molecular diversity.

## 2. Methods

### 2.1 Ethics statement

Written informed consents were obtained for all patients from the ANRS HIV-2 cohort at the time of inclusion in the cohort. The ethical committee named Comité de Protection des Personnes Ile-de-France XI (CPP IDF XI) approved the cohort and its sub-studies on January 2002. All related data are anonymized before collection and analysis.

### 2.2 Patients and sequences data

All HIV-2 group A and B sequences generated for drug resistance monitoring or research purpose within the French ANRS CO5 HIV-2 cohort since 1994 were analysed. These comprised a total of 444 partial *pol* (encompassing the full protease (PR) and partial reverse transcriptase (RT) genes; 1,350 nt) sequences from 291 patients (including 155 patients not present in any of the other genes’ datasets), 129 partial *env* (V3 loop; 525 nt) sequences from 106 unique patients (including 20 not present in other datasets), 152 complete *vif* (655 nt) sequences from 147 patients (including 33 not present in other datasets) and 74 long terminal repeats (LTR; 526 nt) sequences from 74 patients (including 53 not present in other datasets). All publicly available HIV-2 sequences sampled outside of France were retrieved from GenBank and included in the analysis. Viral clones, duplicates, lab strains and sequential sequencing were excluded, leading to the addition of 221, 377, 18 and 63 sequences from unique patients for *pol*, *env*, *vif* and LTR*s*, respectively. All SIV publicly available sequences retrieved from *Sooty mangabey* (SIVsmm) or *Macaca mulatta* (SIVmac) were also included for all corresponding genes (*n* = 63). To complete the analysis and confirm the observation based on separate genes, the dataset of near full genome sequences was retrieved from the Los Alamos National Laboratory. This dataset, extracted in January 2019, represented the fullest spectrum in 2017 of available HIV-2 near full genome sequences and included 30 HIV-2 group A, 7 HIV-2 group B, 4 non-A non-B HIV-2 groups, and 27 SIV sequences. Sequences from recombinant HIV-2 strains were excluded from this analysis (*n* = 4). The sequences from the ANRS CO5 HIV-2 cohort were associated to clinical, epidemiological, virological and immunological data for all included patients, including country of birth, sampling date and HIV-2 group. The country of sampling and sampling date was recorded for the publicly available sequences when available.

### 2.3 Alignments and data selection

For each genetic region, multiple sequence alignments were recursively constructed with Clustal Omega (Sievers and Higgins 2014) and Muscle (Edgar 2004), then manually improved in AliView 1.17.1 (Larsson 2014). All alignment were tested for the presence of recombinants using the package RDP4 under default settings (Martin et al. 2015). For phylogeographic analyses, only the first available sequence for each patient with a known sampling date and country of birth were included, even if the country of sampling was unknown. For patients among the French ANRS CO5 HIV-2 cohort, as all of them were sampled in France, the country of birth was used for geographic inference. The classic approach for phylogeographic analysis is to use the country of sampling for the viral location and origin. This is done under the assumption that the patient has been infected, and will infect further people, at the sampling place. However, this approach presents a limitation in the context of our study. Indeed, only a low number of patients with available sequences sampled across all countries of Western Africa and we add here many sequences sampled in France. If the sampling location was used to calibrate the phylogeographic reconstruction, France would be over-represented and lead to erroneous results (e.g. a French origin of the epidemic). Since nearly all French cohort samples were collected from immigrants, we believe it is fit, in the context of the current study, to use the country of origin of the sampled individual rather than the sampling location of the virus. Country as origin was therefore used as a surrogate of a country on infection. This assumption was supported by the very high proportion of patients born in West Africa among the French ANRS CO5 HIV-2 cohort. As only the first available sequence for each patient was used, a very few resistance-associated mutations were expected and observed. As natural polymorphisms are also present at these positions, we kept them in our phylogeographic reconstruction. However, the reconstruction was also performed without those positions to ensure their potential influence (cf. Supplementary Fig. S2).

### 2.4 Maximum likelihood phylogenetic reconstructions

Maximum likelihood phylogenetic trees were reconstructed using PhyML 3.0 (Guindon et al. 2010), under the GTR-G(4) nucleotide substitution model (as selected by the model testing algorithm jModelTest (Darriba et al. 2012)). Branch support was assessed by the bootstrap method with 1,000 replicates. Pairwise genetic distances between specific clades were calculated with HyPhy 2.2.4 under the GTR-G(4) evolutionary model (Pond, Frost, and Muse. 2005). Phylogenies were edited using FigTree 1.4.4 (http://tree.bio.ed.ac.uk/software/figtree/).

### 2.5 Phylogeographic analyses

Phylogeographic analyses were performed separately for HIV-2 group A and B, including all *pol* sequences with available country of birth and sampling date from the ANRS CO5 HIV-2 cohort or available country and date of sampling from publicly available sequence databases. For *env*, *vif*, or LTR, sequences with available geographic information and sampling dates were too scarce among the French ANRS CO5 HIV-2 cohort or publicly available sequences of the corresponding gene fragments to allow strongly supported phylogeographic analyses.

A preliminary analysis using TempEst was conducted to check the absence of sequence presenting obvious incorrect sampling date or immanent abnormalities in comparison with the global phylogenetic signal of the dataset. Phylogeographic reconstructions were performed using BEAST 1.8.4 (Drummond and Rambaut 2007) with the Gaussian Markov Random Field (GMRF) Bayesian skyride coalescent model (Minin, Bloomquist, and Suchard. 2008), the GTR-G model of nucleotide substitutions, a discrete symmetric trait substitution model and with either a strict or relaxed molecular clock model. The best-fitting molecular clock model was identified by marginal likelihood estimation (Baele et al. 2012, 2013). We ran two sets of 50 million generations each, sampling every 10,000th generations. Parameter estimations were deemed satisfactory when their effective sampling size was above 200 in Tracer 1.6. After discarding 10 per cent burn-in, combined files were produced using LogCombiner. The maximum clade credibility (MCC) tree generated with TreeAnnotator was then annotated in FigTree v1.4.3. Changes in effective population size over time were estimated from the time tree distribution generated by BEAST, using Tracer 1.6.

To test the potential links observed between group A lineages and their geographical distribution allowing to characterize potential founder effects, we used the tool BaTS, a software using a Bayesian Markov-Chain Monte Carlo approach to the investigate phylogeny-trait correlations while taking into account the uncertainty arising from phylogenetic error (Parker, Rambaut, and Pybus 2008). Briefly, this method compares a posterior distribution of trees to a null distribution of 100 trait-randomized trees here selected from Group A sequences Bayesian reconstruction as previously described. Group A sequences were tested to assess if the clustering in the tree is associated with the two geographical regions identified in our previous analyses (i.e. inland and coastal Wester countries). The overall statistical significance was determined by estimating the parsimony score (PS) and association index (AI) metrics, where the null hypothesis is that clustering by geographic location is not more than that expected by chance. In addition, the maximum clade (MC) size metric was used to compare the strength of clustering at each location by calculating the expected (null) and the observed mean clade size from each study location. A significance level of 0.05 was used in all cases. The PS, AI, and MC statistics were computed for a null distribution with 1,000 replicates.

## 3. Results

### 3.1 Phylogenetic analyses

**Figure 1.**
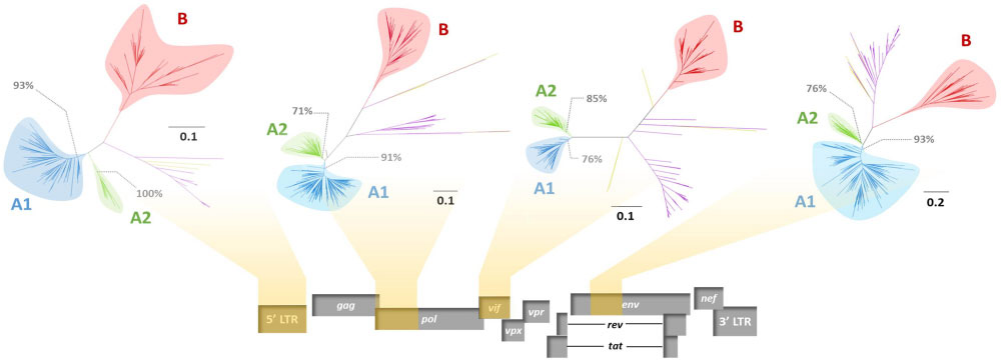
Unrooted phylogenetic trees obtained by approximate maximum likelihood available based on four genomic regions: LTR, *pol*, *vif* and *env*. SIV sequences are indicated in purple and non-A non-B HIV-2 sequences are indicated in yellow. Branch lengths represent the number of nucleotide substitutions per sites, as indicated on the scale. Branch support values are indicated in grey for the A1 and A2 most recent common ancestor corresponding nodes.

**Figure 2.**
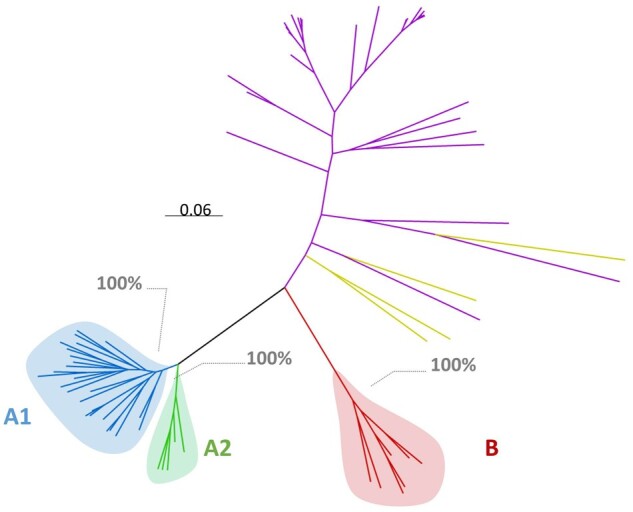
Unrooted approximate maximum likelihood phylogenetic tree of 68 HIV-2 near-full genome sequences. SIV sequences (in purple) and non-A non-B HIV-2 sequences (in yellow) were included as outgroup. Branch lengths represent the number of nucleotide substitutions per sites, as indicated on the scale. Branch support values are indicated in grey for the A1 and A2 most recent common ancestor corresponding nodes.

The phylogenies of the four studied HIV-2 genomic regions are shown in Figs. 1 and 2. HIV-2 group A consistently formed two distinct clades, herein called A1 and A2. These two clades were strongly supported on each tree (branch support ranging from 76% to 100%) and were also present in the phylogeny reconstructed from full genomes. The clades diverge deep in the Group A lineage, suggesting a split soon after the introduction of the virus in the human population. Among patients with sequences available for several genes, only 17 (9%) presented inconsistent clade assignments across trees.

**Figure 3.**
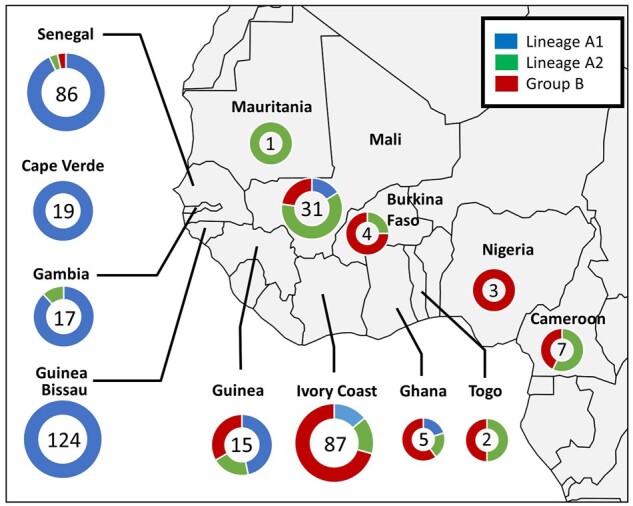
Geographical distribution of HIV-2 clades observed in Western Africa, the epicentre of the HIV-2 epidemic, using the country of origin of all patients included in the French ANRS CO5 HIV-2 cohort and the country of sampling for publicly available sequences obtained in this area. The number of patients per country is given in each corresponding circle. Proportion of HIV-2 A1, A2, and B lineages is indicated by the blue, green, and red colours within each circle, respectively.

The geographical distribution of HIV-2 lineages in the study cohort is shown in Fig. 3. The HIV-2 epidemic in coastal Western African countries (i.e. Cape Verde, Senegal, Gambia, Guinea Bissau and Guinea) was dominated by lineage A1 with 245/261 (94%), compared with 8/261 (3%) A2 and 8/261 (3%) B viruses. Inversely, inland western countries (i.e. Mali and Burkina-Faso) and coastal Southern countries (i.e. Ivory Coast, Ghana, Togo, Nigeria and Cameroon) were dominated by A2 and B lineages with 18/140 (13%), 41/140 (29%) and 81/140 (58%) of A1, A2 and B viruses, respectively. Outside of Western Africa, the sequences sampled in Portugal were exclusively of A1 viruses (154/154, 100%) while a mixture of A1 (49/114; 43%), A2 (29/114; 25%) and B sequences (32/114; 28%) were found in patients born in France. The differences observed in lineage distribution among the coastal Western countries and inland Western countries geographic areas were statistically significant (chi-square test; *P* < 0.001). These distinct geographic distributions were confirmed by the phylogeny-trait association test, which revealed a statistically significant structuring by these geographical area (*P*-values at 0.003 and 0.02 for the MC statistics of inland and coastal Western countries, respectively, cf. Supplementary Table S3).

A1 and A2 HIV-2 lineages presented a median genetic distance of 0.13 [interquartile range = 0.13–0.14], 0.12 [0.11–0.14], 0.12 [0.11–0.13], 0.16 [0.12–0.18], and 0.18 [0.16–0.21] substitution/nucleotide for near full genome, *pol*, *env*, *vif*, and LTR, respectively. Detailed genetic distance distribution within and between HIV-2 A clades are depicted in Supplementary Fig. S1. The numbers of included sequences falling into each of the three main HIV-2 lineages (i.e. A1, A2 and B) for both French ANRS CO5 HIV-2 cohort and non-French publicly available sequences are depicted on Supplementary Tables S1 and S2. It highlights the very low number of A2 and B sequences among non-French publicly available sequences.

### 3.2 Clinical status across HIV-2 A subclades

The characteristics of the 264 included patients from the French ANRS CO5 HIV-2 cohort with available clinical data are depicted in Table 1. Among these patients, age at inclusion, sex, mode of contamination, viral loads and nadir of CD4 were available for 241, 241, 241, 104, and 240 patients, respectively.

**Table 1.** Main characteristics of all patients from the ANRS CO5 cohort included in this study overall and according to the virological clade of their viruses.

	Total	A1	A2	B	*P*
*n*	264	93	68	103	
Sex (% female)	54.1	56.8	55.0	51.1	0.72
Median age at inclusion [IQR]	41.0 [34.2–48.3]	42.7 [37.2–50.7]	42.0 [35.3–50.6]	39.0 [33.4–45.4]	0.01
Mode of contamination					0.18
Heterosexual	200 (88.9%)	70 (86.4%)	55 (94.8%)	75 (87.2%)	
MSM	3 (1.3%)	2 (2.5%)	1 (1.7%)	0 (0%)	
Mother to child	4 (1.8%)	2 (2.5%)	1 (1.7%)	1 (1.2%)	
Transfusion	18 (8.0%)	7 (8.6%)	1 (1.7%)	10 (11.6%)	
CDC stage at inclusion					0.03
A/B	199 (85.4%)	71 (83.5%)	44 (77.2%)	84 (92.3%)	
C	34 (14.6%)	14 (16.5%)	13 (22.8%)	7 (7.7%)	
Aviremic patients	43.1%	46.3%	34.5%	46.2%	0.59
Median VL [IQR]	1071 [219–4614]	1098 [175–8462]	2018 [274–5746]	853 [527–2120]	0.90
Median nadir of CD4 [IQR]	231 [101–431]	247 [153–458]	203 [62–378]	226 [77–427]	0.25

IQR, interquartile range.

No difference in immune-virological characteristics was observed between A1-, A2-, or B-infected individuals. Only the age and the CDC clinical stage of the disease were statistically different among the three clades with younger and less advanced diseases among patients infected with HIV-2 group B than in A1 or A2 lineages (*P* = 0.008 and 0.02, respectively). Viral load and nadir of CD4 were not statistically different across groups even if the A2 clade tended to a lower representation of spontaneously aviremic patients, to higher viral load at diagnosis among viremic patients and to lower nadir of CD4 compared to the A1 clade (Table 1). As the three clades are distinctly distributed among countries, we cannot assess if the clinical and demographic differences are related to the place of birth or to the viral lineage.

### 3.3 Phylogeographic reconstruction of HIV-2 clades early dispersal

**Figure 4.**
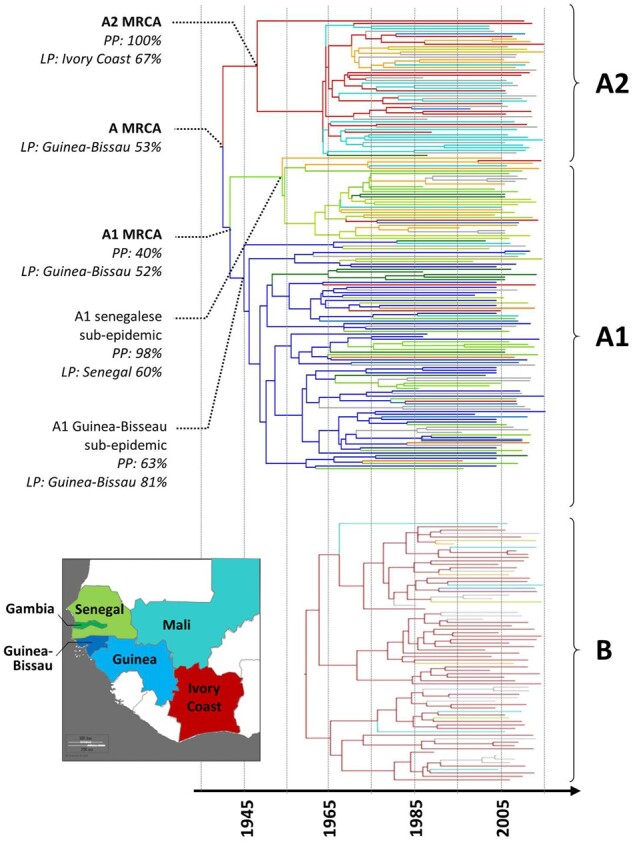
Bayesian MCC tree obtained for HIV-2 groups A and B using pol sequences. These trees are time-scaled, with branch lengths expressed as calendar years, and the colour of each branches depict the most probable location of the corresponding ancestor. The colour code for Western Africa is depicted on the map: dark blue is for Guinea-Bissau, blue for Guinea, Green for Senegal, dark green for Gambia, red is for Ivory Coast and turquoise for Mali. France is coloured in orange, all the other countries are coloured in grey. For the nodes of the main most recent common ancestors (MRCA), the posterior probability (PP) and the most probable location with its location state probability (LP) are given.

**Figure 5.**
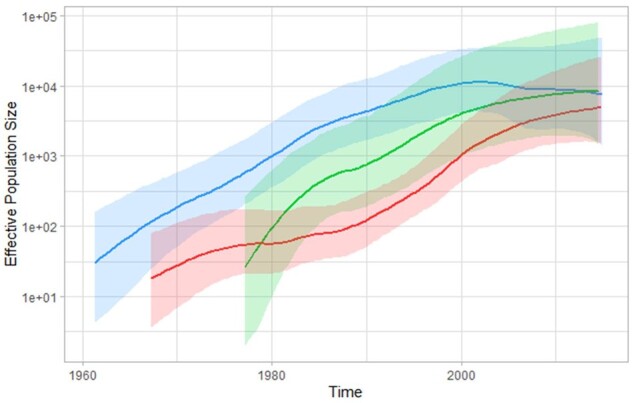
Changes in effective population size over time for the three main HIV-2 lineages. A1 is depicted in blue, A2 in green and B in red.

All HIV-2 *pol* sequences for which known time of sampling and patient's country of birth were retrieved from public databases (*n* = 49 and 8 for groups A and B, respectively) and from the ANRS CO5 HIV-2 cohort database (*n* = 125 and 68 for groups A and B, respectively). The best-fit model combination was found to be the GTR-G nucleotide substitution model with GMRF Bayesian skyride coalescent model and a lognormal relaxed molecular clock. The Bayesian MCC trees obtained from these sequences for both groups A and B are shown in Fig. 4. Estimated changes in effective population size over the time spanned by the dated phylogeny are showed, for each lineage, in Fig. 5. The evolution rates were estimated at 7.70 × 10^−3^ (95% higher probability density (HPD): 4.43 × 10^−3^; 1.20 × 10^−2^) and 7.67 × 10^−3^ (95% HPD: 4.51 × 10^−3^; 1.12 × 10^−2^) substitution per year per site for HIV-2 Groups A and B, respectively.

The divergence between the A1 and A2 lineages occurred at the very beginning of the epidemic in 1940 (95% HPD: 1935–53). The most probable location state at the node representing lineage divergence in our phylogeographic reconstruction is Guinea-Bissau (location state probability at 0.53, higher than Senegal at 0.26 and Ivory Coast at 0.08). The A1 lineage, mainly present in patients born in Senegal, Gambia, Guinea-Bissau and Guinea, experienced an early diversification in 1942 (1937–1958) with two distinct sub-epidemics: the main one emerging in Guinea-Bissau (location state probability at 81%) and a second one in Senegal (location state probability at 60%, before France at 31%). The Senegalese epidemic presented a latter diversification, starting at 1954 (1946–1965), than the Guinea-Bissau epidemic which presents a quite regular diversification starting at 1945 (1940–1957). Several subsequent transmission events from Guinea-Bissau to Senegal were also observed in the late 60s.

HIV-2 group B started to diverge in 1961 (1952–70) and appeared as mostly restricted to Ivory Coast as all deep tree nodes are located in this country (location state probability >99%). All the other countries are dispersed across the whole group B phylogenetic reconstruction and no important unique transmission event are identified from Ivory Coast to any other country.

## 4. Discussion

Using phylogenetic approaches with all HIV-2 sequences available to date from the French ANRS HIV-2 cohort and publicly available databases, we provided several new insights about HIV-2 molecular epidemiology. We observed the existence of two distinct lineages within HIV-2 group A, herein called A1 and A2, related to distinct geographical origins but non-significant immuno-virological differences. We also described the early epidemic spreading patterns in West Africa and recent epidemiological trends, especially in Senegal, Mali and Ivory Coast where sequence and recent prevalence data are lacking. Our phylogeographic reconstructions estimated the HIV-2 group A tMRCA around 1940 (95% HPD: 1935–53), in range with previous estimates at 1940 ± 16 (Lemey et al. 2003) and 1938 [1928–1947] (Faria et al. 2012). In our dataset, the group B tMRCA among human population was estimated to 1961 (1952–70). Very few papers have previously attempted to date this for HIV-2 group B, but our result is sensibly later than previous divergence time estimates done by Wertheim et al. between 1914 (1868–1955) and 1937 (1914–58) (Wertheim and Worobey, 2009). To confirm our findings, we also estimated the HIV-2 group B tMRCA in our dataset without geographic trait analysis and using Skygrid and Skyline coalescent models. Both provided similar tMRCA estimations than in our phylogeographic analysis at 1955 (1939–66) and 1955 (1938–68). Thus, if HIV-2 group B raised latter than group A in both works, the precise emergence time estimation may still need to be refined in the future using other sequence datasets. The distribution of HIV-2 subtypes across Western Africa observed with all included sequences in this study (i.e. all retained sequences for LTR, PR+RT region of *pol*, *vif*, and V3 loop region of *env*, along with available geographic origins) was in perfect line with the previously described distribution using the subtype and geographic information of all sequences deposited on GenBank and previously described (Visseaux et al. 2016). However, the current work confirms the existence of two HIV-2 A subclades, A1 and A2, and their differential geographic distribution.

These two HIV-2 A sub-clades were consistent across all maximum likelihood trees. Despite overlaps, especially for pol or env, these subclades presented a non-negligible genetic distance for all genes or the whole genome analysis, with statistically larger between A1 and A2 that within A1 or A2 genetic distances (*P* < 0.001 for all comparisons for all genes, cf. Supplementary Fig. S1). The different geographic distributions are suggestive of early founder effects but also different evolutions of the epidemic. HIV-2 A1 tMRCA is 1942 (1937–58), this lineage is widely present among non-French publicly available sequences (cf. Supplementary Table S1) and linked to Guinea-Bissau and surrounding countries by both the geographic distribution of patients (cf. Fig. 3) and the phylogeographic reconstruction (cf. Fig. 4). On the other hand, HIV-2 A2 appeared as linked to French former colonies, Ivory Coast and Mali, explaining why samples from these countries are more abundant in the French ANRS CO5 HIV-2 cohort than in public resources. This under-representation of the A2 lineage in public sequence datasets not sampled in France may explain why the two A sub-lineages were not previously strongly characterized but only mentioned, to our knowledge, in a single French study conducted on thirty patients (Damond et al. 2001). HIV-2 A2 presented a latter diversification than HIV-2 A1 with a tMRCA in 1954 (1948–64). However, this time is mostly driven by a single sequence from an Ivorian patient belonging to the French ANRS CO5 HIV-2 cohort and branching quite deeply into the tree compared to other sequences. Most of A2 diversification occurred even latter, starting at 1963 (1957–69).

We also identified, within the A1 lineage, a very early divergence of HIV-2 A1 between Senegal and Guinea-Bissau. This divergence was estimated 1942 (1937–58) with our dataset, before the time of Guinea-Bissau independence war (1963–74), suggesting the possibility of an initial introduction of HIV-2 A from Ivory Coast to Guinea-Bissau or Senegal before the large spread of the disease in the neighbouring area. This hypothesis can make sense as (i) it has been previously established that Ivory Coast should be the birthplace of the HIV-2 epidemic as both groups A and B are closely related to the SIV from the Taï Forest in this country (Santiago et al. 2005) and (ii) Senegal and Ivory-coast are both former French colonies and, thus, shared more traveling connections than with Guinea-Bissau, a former Portuguese colony. However, this possible scenario has to be taken with caution. Indeed, it can be suggested from the observed tree topology but is in contradiction with the most probable country attributed to the root and the A1 most recent common ancestor (MRCA) in our model as both are attributed to Guinea-Bissau. In a previous phylogeographic reconstruction of HIV-2 group A, the root was also attributed to Guinea-Bissau and not Ivory Coast (Faria et al. 2012). In this study, as in the current work, this can be the result of the heterogeneous sampling scheme across West African countries that may biased the geographic attribution particularly among these two nodes, the root and A1 MRCA, the deepest of our reconstruction. Thus, two scenarios could be drawn here, and we cannot exclude one of them: (i) a HIV-2 group A birthplace in Guinea-Bissau and further transmission to Ivory-Coast and Senegal, which is suggested by the location state reconstruction of the two deepest node of our tree, or (ii) a HIV-2 group A birth place in Ivory-Coast with further transmission in the area and a transmission event to Senegal and, then Guinea-Bissau. The latter may be supported by the tree topology, the Taï forest location of HIV-2 A SIVs ancestor and the colonial connection at the time. This uncertainty regarding these two scenarios is illustrated by the phylogeographic reconstruction obtained when stripping drug resistance mutations that provided a tree better supporting the former Guinea-Bissau scenario, even if slightly less strongly supported than our first reconstruction (cf. Supplementary Fig. S2). Unfortunately, we do not dispose of sufficient sequences and epidemiological data over time to fully assess the potential bias of the heterogeneous sampling scheme over West Africa, even if the current dataset may provide a better representation of the epidemic in Ivory-Coast or Mali than in previous studies. The strong diversification of the HIV-2 lineage A1 in the 60s and 70s re-enforces the hypothesis of an important increase of HIV-2 incidence in Guinea-Bissau and surrounding countries during the Guinea-Bissau independence war (1963–74), as previously described (Lemey et al. 2003). The role of the war in the spread of HIV-2 in the area can be discussed as HIV-2 A2 and B lineages were also able to expend at large rates without a war event, some other causes may also be at the origin of HIV-2 A1 spread in the area.

The HIV-2 A2 lineage, centred on Mali and Ivory Coast showed population dynamics slightly different from A1 with a latter initial increase in the early 80s. Group B, mostly centred on Ivory-Coast presented an even latter initial increase in the early 90s. At the beginning of the 2000’s, a slight decrease of effective population size is observed for the A1 lineage while HIV-2 A2 and B appears to reach a plateau (cf. Fig. 5). These observations highlight that if HIV-2 is decreasing in Guinea-Bissau, A2 and B lineages may remain more active in other part of Western Africa. There was no war event in the 80’s and 90’s that may be pointed out as responsible of disease spread acceleration. However, we can note a large economic crisis observed in those countries, and particularly Ivory-Coast, during this period that may have play some role due to strong sociological and behaviours changes at the time (Ghai and Alcántara 1990).

Our estimates may be partially biased by the sampling heterogeneity of our cohort. Due to the lack of precise epidemiological data on HIV-2 epidemiology in Western Africa, some geographical regions of epidemiological relevance may be poorly or not represented in our dataset. This could bias the phylogenetic reconstructions by missing parts of HIV-2 diversity or overrepresenting others. To reduce this bias to a minimum, publicly available sequences were included when possible. This augmented dataset allowed an overall good representation of all African countries afflicted by the HIV-2 epidemic (8). Despite this limitation, the addition of all available French ANRS CO5 HIV-2 cohort sequences allows a better display of HIV-2 lineages from inland Western Africa countries and coastal Southern Africa countries, rare among publicly available sequences, and, thus of A2 and B lineages evolution. To help assessing the strength of our conclusion, we assessed the sampling heterogeneity in our phylogeographic reconstruction using a downsampling approach. Two downsampled datasets were constructed: (i) a structured dataset (all countries with more than 10 sequences were randomly downsampled to 10 available sequences, all the other countries sequences were kept for phylogenetic reconstruction); and (ii) a random dataset (half of the sequences were randomly chosen independently of their geographic origins). The obtained geographic distributions of sequences in those datasets are presented in Supplementary Table S4. We obtained in both cases a similar phylogeographic reconstruction, presented and discussed in Supplementary Fig. S3.

Another limitation of our study is the use of the patient’s country of birth as an indicator of the origin of the viruses infecting them. This was done under the assumption that most of these infections were acquired abroad or transmitted from patients infected abroad. This assumption is supported by the characteristics of HIV-2 infections, including a slower evolution to AIDS than HIV-1 (Marlink et al. 1994; Matheron et al. 1997; Poulsen et al. 1997; Drylewicz et al. 2008; Thiébaut et al. 2011), low transmission rates (Kanki et al. 1994; Gomes et al. 2003; Gottlieb et al. 2006) and is in line with the large number of patients born in West Africa among patients included in the French ANRS CO5 HIV-2 cohort (76% of patients included in this study) but may obviously not be true for the totality of our patients. Using the country of sampling for patients sampled in France induced a too large number of sequences coming from France and strongly biased the geographic reconstruction.

This study shed light on the early evolutionary and epidemiological history HIV-2, characterizing two well-separated clades among the HIV-2 A group and their migration pattern in Western Africa in the early epidemic. We show that the diversification of these sub-lineages occurred soon after the introduction of the ancestral HIV-2 A virus into the human population, and likely result of founder effects driven by the social-economical context of the time. These two clades also present small but non negligible genetic distances and distinct geographical origins and, therefore, may be proposed as the first subtypes among the HIV-2 classification. Such distinction should be helpful as these lineages presents distinct past epidemiological evolutions that will be useful to follow in future epidemiological studies.

## Supplementary Material

veab024_Supplementary_Data
