# Supplementary material for: HIV-2 diversity displays two clades within group A with distinct geographical distribution and evolution
Source: Virus Evol. 2021 Mar 16;7(1):veab024. doi: 10.1093/ve/veab024 (PMC8377049; doi:10.1093/ve/veab024)
Supplement: veab024_Supplementary_Data [file veab024_supplementary_data.docx]

**Supplementary Table 1. Distribution of HIV-2 clades across all studied genomic regions and all included sequences, sequences from the French ANRS CO5 HIV-2 cohort or from non-French publicly available sequences.**

| **Genome region** | **Total** | **A1** | **A2** | **B** | **Other groups** |
| --- | --- | --- | --- | --- | --- |
| ***Protease and reverse transcriptase genes*** | **665 (100%)** | **366 (55%)** | **138 (20.8%)** | **156 (23.5%)** | **5 (0.8%)** |
| ANRS CO5 cohort sequences | 444 (100%) | 190 (42.8%) | 118 (26.6%) | 136 (30.6%) | 0 (0%) |
| Publicly available sequences | 221 (100%) | 176 (79.6%) | 20 (9%) | 20 (9%) | 5 (2.3%) |
| ***Envelope gene*** | **506 (100%)** | **362 (71.5%)** | **44 (8.7%)** | **87 (17.2%)** | **13 (2.6%)** |
| ANRS CO5 cohort sequences | 129 (100%) | 54 (41.9%) | 19 (14.7%) | 56 (43.4%) | 0 (0%) |
| Publicly available sequences | 377 (100%) | 308 (81.7%) | 31 (8.2%) | 25 (6.6%) | 13 (3.4%) |
| ***Vif gene*** | **178 (100%)** | **58 (32.6%)** | **44 (24.7%)** | **72 (40.4%)** | **4 (2.2%)** |
| ANRS CO5 cohort sequences | 152 (100%) | 47 (30.9%) | 40 (26.3%) | 65 (42.8%) | 0 (0%) |
| Publicly available sequences | 18 (100%) | 5 (27.8%) | 4 (22.2%) | 5 (27.8%) | 4 (22.2%) |
| ***LTR*** | **137 (100%)** | **69 (50.4%)** | **14 (10.2%)** | **50 (36.5%)** | **4 (2.9%)** |
| ANRS CO5 cohort sequences | 74 (100%) | 22 (29.7%) | 10 (13.5%) | 42 (56.8%) | 0 (0%) |
| Publicly available sequences | 63 (100%) | 47 (74.6%) | 4 (6.3%) | 8 (12.7%) | 4 (6.3%) |

**Supplementary Table 2. Distribution of HIV-2 sequences with available sampling date, country of birth (for French sequences) or country of sampling (for public sequences), included in the phylogeographic reconstruction.**

|  | **Total** | **French sequences** | **Public sequences** |
| --- | --- | --- | --- |
| *Ivory Coast* | 63 | 60 | 3 |
| *Senegal* | 35 | 24 | 11 |
| *Guinea-Bissau* | 29 | 8 | 21 |
| *Mali* | 26 | 26 | 0 |
| *France* | 24 | 24 | 0 |
| *Guinea* | 12 | 12 | 0 |
| *Gambia* | 12 | 3 | 9 |
| *Cap Verde* | 8 | 8 | 0 |
| *Portugal* | 6 | 6 | 0 |
| *Cameroon* | 5 | 4 | 1 |
| *Ghana* | 5 | 2 | 3 |
| *Burkina-Faso* | 4 | 4 | 0 |
| *Mauritania* | 3 | 3 | 0 |
| *Nigeria* | 3 | 1 | 2 |
| *Other* | 15 | 8 | 7 |

**Supplementary Table 3. Statistics obtained with BaTS when performing phylogeny trait association analysis of the distribution of inland and coastal Western Africa countries within the group A lineage.**

| **Statistic** | **observed mean** | **lower  95% CI** | **upper  95% CI** | **null mean** | **lower  95% CI** | **upper  95% CI** | **signifi-cance** |
| --- | --- | --- | --- | --- | --- | --- | --- |
| Association index | 3.33 | 2.83 | 3.83 | 6.67 | 5.53 | 7.9 | 0 |
| Parcimony score | 15.78 | 15 | 16 | 36.72 | 33.55 | 39.6 | 0 |
| MC (state 0 - inland countries) | 6.94 | 5 | 11 | 2.73 | 2.02 | 3.91 | 0.003 |
| MC (state 1 - Coastal countries) | 14.64 | 12 | 25 | 6.42 | 4.56 | 9.05 | 0.017 |

**Supplementary Table 4. Geographic distribution of HIV-2 sequences in the full dataset and the two downsampled datasets.**

|  | **Initial dataset** | **Structure downsampling** | **Random downsampling** |
| --- | --- | --- | --- |
| *Sengal* | 33 | 10 | 23 |
| *Guinea-Bissau* | 29 | 10 | 18 |
| *Mali* | 20 | 10 | 8 |
| *France* | 18 | 10 | 8 |
| *Ivory Coast* | 16 | 10 | 11 |
| *Gambia* | 12 | 10 | 5 |
| *Guinea* | 10 | 10 | 5 |
| *Cap-Verde* | 8 | 8 | 6 |
| *Portugal* | 5 | 5 | 1 |
| *Cameroon* | 3 | 3 | 3 |
| *Ghana* | 3 | 3 | 2 |
| *Other* | 14 | 14 | 7 |

**Supplementary Figure 1.** **Distribution of genetic distances observed within and between HIV-2 A clades.** Results are depicted according the studied genomic regions: near full genome sequences (A), *pol* gene region encompassing the protease and the reverse transcriptase (B), *vif* gene (C), *env* area encompassing the V3 loop (D) and the LTR (E).


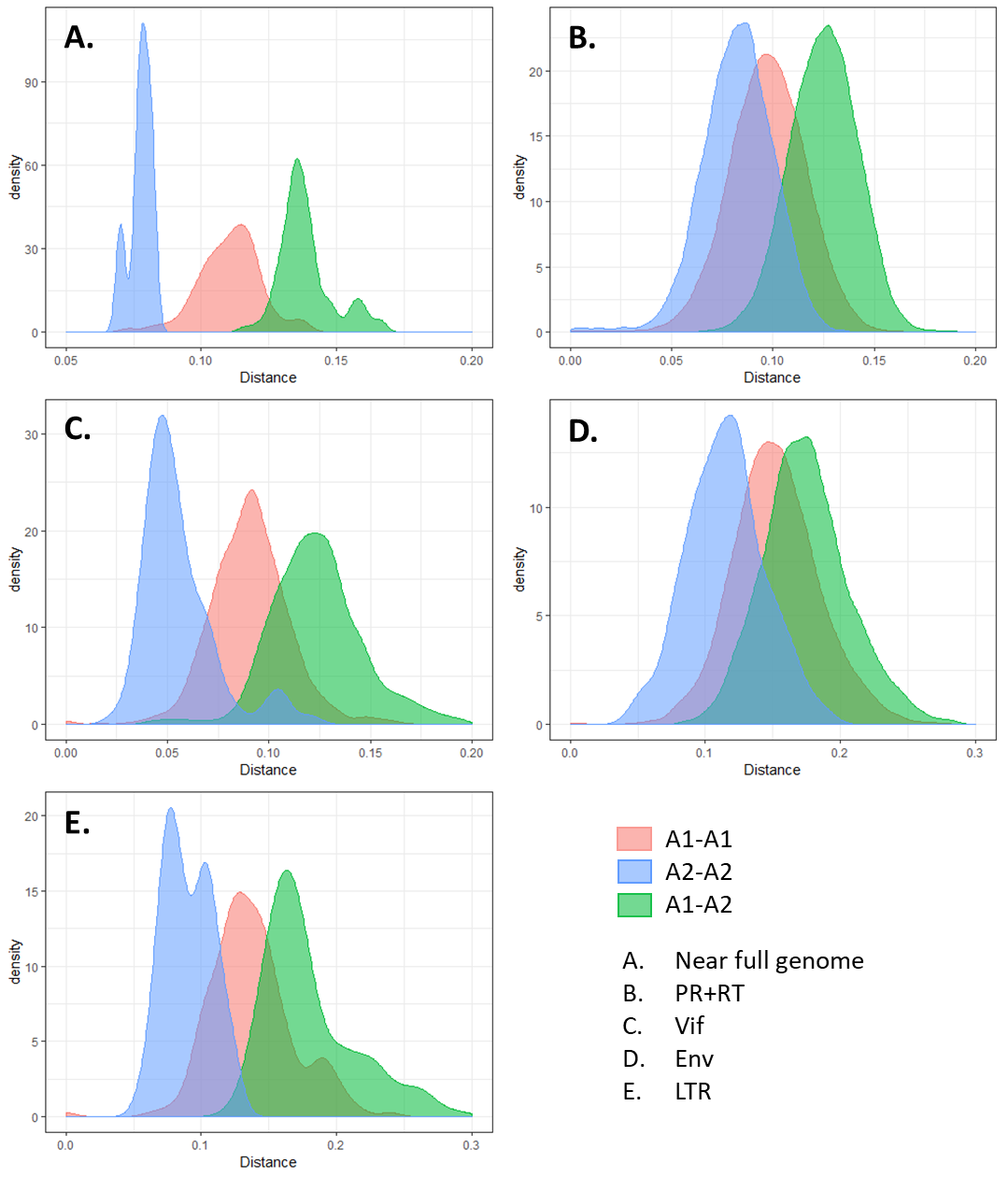


**Supplementary Figure 2.** **Bayesian maximum clade credibility tree obtained for HIV-2 group A using pol sequences without drug resistance mutations.** The tree is time-scaled, with branch lengths expressed as calendar years, and the colour of each branches depict the most probable location of the corresponding ancestor. The colour code for Western Africa is depicted on the map: dark blue is for Guinea-Bissau, blue for Guinea, Green for Senegal, dark green for Gambia, red is for Ivory Coast and turquoise for Mali. France is coloured in orange, all the other countries are coloured in grey. For the nodes of the main most recent common ancestors (MRCA), the posterior probability (PP) and the most probable location with its location state probability (LP) are given. Potential recent transmission clusters were previously identified on a maximum likelihood tree, constructed as depicted in method section, as sequences presenting a genetic distance <4.5 subst. per 100 nucleotide and a branch support value above 95%.


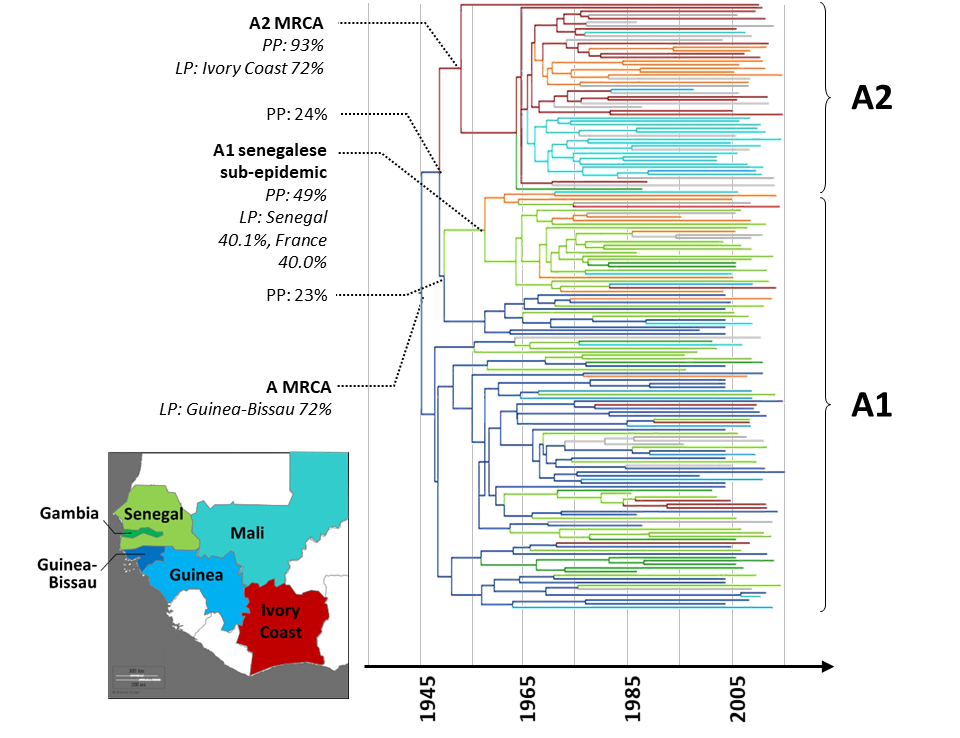


**Supplementary Figure 3.** **Bayesian maximum clade credibility trees obtained for HIV-2 group A using pol sequences with the two downsampled datasets.** The tree is time-scaled, with branch lengths expressed as calendar years, and the colour of each branches depict the most probable location of the corresponding ancestor. The colour code for Western Africa is depicted on the map: dark blue is for Guinea-Bissau, blue for Guinea, Green for Senegal, dark green for Gambia, red is for Ivory Coast and turquoise for Mali. France is coloured in orange, all the other countries are coloured in grey. For the nodes of the main most recent common ancestors (MRCA), the posterior probability (PP) and the most probable location with its location state probability (LP) are given. Potential recent transmission clusters were previously identified on a maximum likelihood tree, constructed as depicted in method section, as sequences presenting a genetic distance <4.5 subst. per 100 nucleotide and a branch support value above 95%. In the structured downsampling dataset, the countries with more than 10 sequences were randomly reduced to 10 sequences whereas, in the random downsampling dataset, one sequence out of 2 was randomly choosen, indenpendantly of its geographic location.


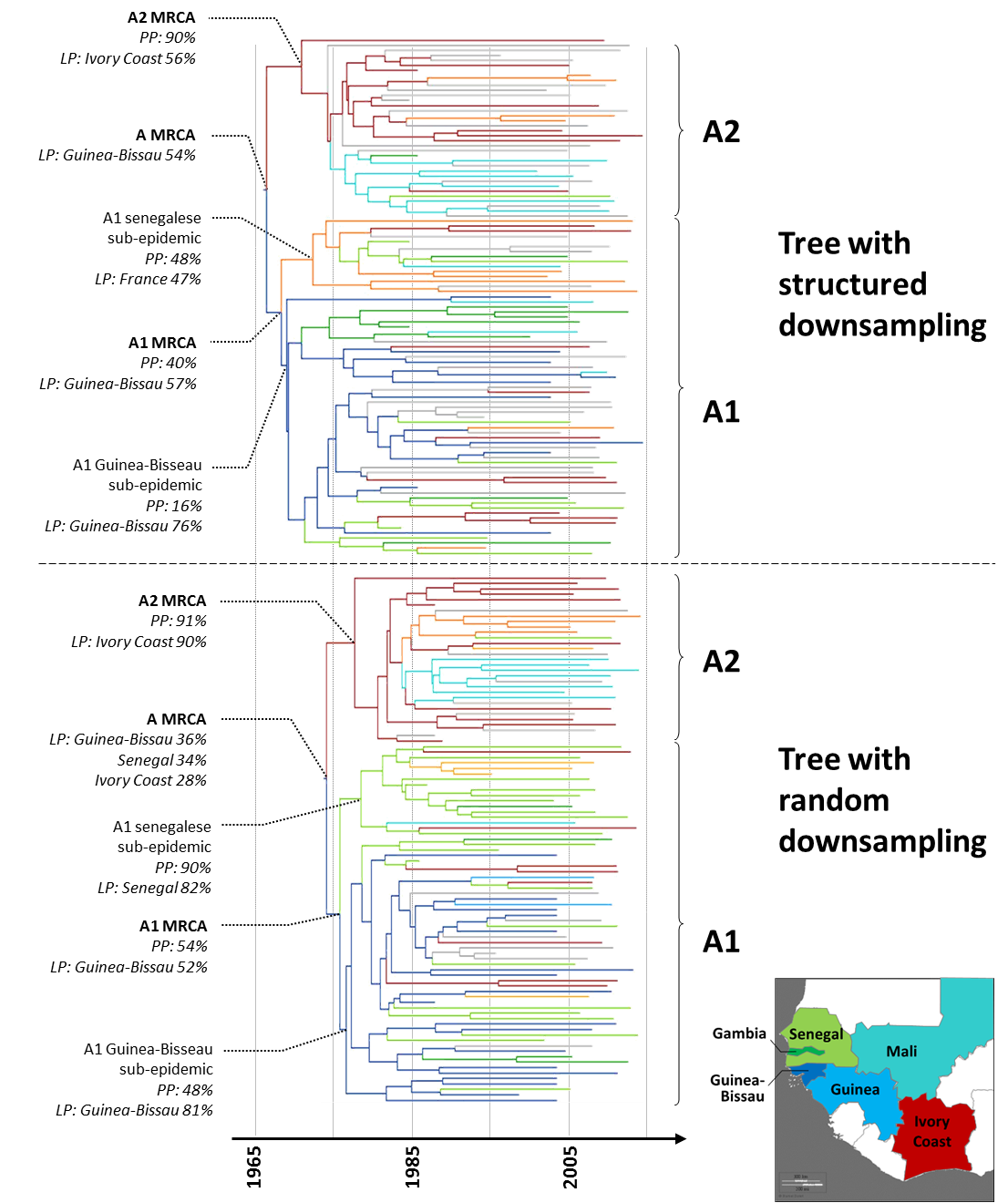


The geographic structures of the two obtained trees presented the same organization than with the full dataset for all deep nodes. The roots were always located in Guinea-Bissau, immediately giving birth to A1 and A2 lineages. The A1 and A2 lineage most recent common ancestors were consistently located in Guinea-Bissau and Ivory Coast, respectively. The A1 MRCA immediately diverged into the A1 Senegalese subepidemic and the Guinea-Bissau subepidemic. However, in the tree obtained with the structured downsampled dataset, the Senegalese A1 MRCA location was attributed to France (location state probability at 47%) and then to Guinea-Bissau (17%), Ivory Coast (17%) and Senegal (16%). This incongruity is explained by the high number of French sequences and the low number of Senegalese sequences obtained in this branch after the downsampling of these two countries. The tree obtained by a fully random downsampling still presented a very strong Senegalese location of the Senegalese subepidemic (location state probability at 82%). Thus, no other discrepancies were observed between the downsampled trees and our initial phylogeographic reconstruction.
